# A randomised control trial of the effectiveness of personalised letters sent subsequent to school dental inspections in increasing registration in unregistered children

**DOI:** 10.1186/1472-6831-9-8

**Published:** 2009-03-12

**Authors:** Chris J Cunningham, Rob Elton, Gail VA Topping

**Affiliations:** 1 NHS Lothian Salaried Primary Care Dental Service, NHS Lothian, Duncan Street Dental Centre, Edinburgh, EH9 1SR, UK; 2 Department. of Community Health Sciences, University of Edinburgh Medical School, Teviot Place, Edinburgh, EH8 9A, UK; 3 Dental Health Services & Research Unit, University of Dundee, Kirsty Semple Way, Dundee, DD2 4BF, UK

## Abstract

**Background:**

Recent studies have cast doubt on the effectiveness and efficiency of school based dental screening programmes in improving dental attendance or improving dental health. In 2002 the National Dental Inspection Programme was introduced in Scotland which categorises children by their dental health and informs parents of the findings via a personalised letter home and encourages dental registration. In addition, epidemiological data for local and national planning purposes is collected. This replaced an earlier school screening system in Lothian where a generic letter urging registration was sent to children who were identified as not being registered with a dentist. The objective of this study is to compare dental registrations rates among unregistered children in these two school inspection systems with a system where letters were sent home but no dental inspection was carried out.

**Methods:**

The study was designed as a single blinded, cluster randomised, controlled trial involving 12,765 12–13-year-old children attending all 65 state Secondary schools in Lothian and Fife during the academic year 2003/4.

After stratifying for school size and range of social deprivation, schools were randomly allocated to one of four groups:

1. 'Traditional' inspection, letter to unregistered children only,

2. Letter sent home to unregistered children only, no inspection,

3. National Dental Inspection Programme, letter to all children,

4. Control group in which the children were neither inspected nor sent a letter.

Dental Registration status was compared at baseline and 3 months post inspection.

**Results:**

The registration levels in both the 'Traditional' screening and the NDIP inspection groups rose 3 months post inspection (14% and 15.8% respectively) but were not significantly different from one another or the control group which rose by 15.8% (p > 0.05). The group who were sent a letter home but were not inspected also has a rise in registration levels of 18.1% which was not significantly different from either of the groups who were inspected or the control group (p > 0.05). The only significant predictors of registration were previous registration (p < 0.05) and within those who previously registered, the length of time since last registration (P < 0.001).

**Conclusion:**

Neither of the two dental inspection methods nor a letter home to unregistered children resulted in a significant rise in registration rates in 12–13-year-olds compared to a control group of children who received no intervention.

## Background

The effectiveness of the school dental screening programme in increasing registration levels has previously been investigated in 3 RCTs [1-3]. In the earlier two studies a significant improvement in registration levels was demonstrated. More recently, a large scale cluster randomised control trial in the North-west of England, did not show any benefit from the screening process either in reducing the amount of untreated dental disease or increasing levels of dental attendance [4]. However none of these studies looked at the effect of a personalised letter home alone or to what degree the combination of the two interventions (dental inspection and personalised letter) affected the proportion registered.

In the academic year 2002/3 a uniform National Dental Inspection Programme (NDIP) was introduced across Scotland [5]. The NDIP involves children in Primary I (around 5-years-old) and VII (around 11-years-old) being inspected under a national, standardised protocol. The children are categorised as high, medium or low risk as determined by the clinical findings. Separate letters are sent to the parents of children in each risk group detailing the clinical findings and encouraging registration with a GDP. No attempt is made to identify the registration status of the child at the time of the inspection, however all are also sent a list of local GDPs.

Prior to this, each NHS Board Community Dental Service (CDS) had run differing screening programmes. The national guidance allowed for dental inspections up to three times in the child's school career. Between NHS Boards there were great variations in inspection frequencies, ages of children inspected, data collection protocols and information sent home. In Lothian and Fife, the CDS had developed links with the local education departments and the Scottish Dental Practice Board in order to electronically match school rolls with dental registration data and thus identify primary and secondary school children who were not registered with a GDP. This system is thought to be unusual in being able to identify named individuals and validate their "official" dental registration status rather than relying on anecdotal parental feedback or hand checking of records. After the clinical inspection these unregistered children were then sent a personalised letter urging them to register with a dentist and also a list of local NHS dentists.

**Figure 1:**
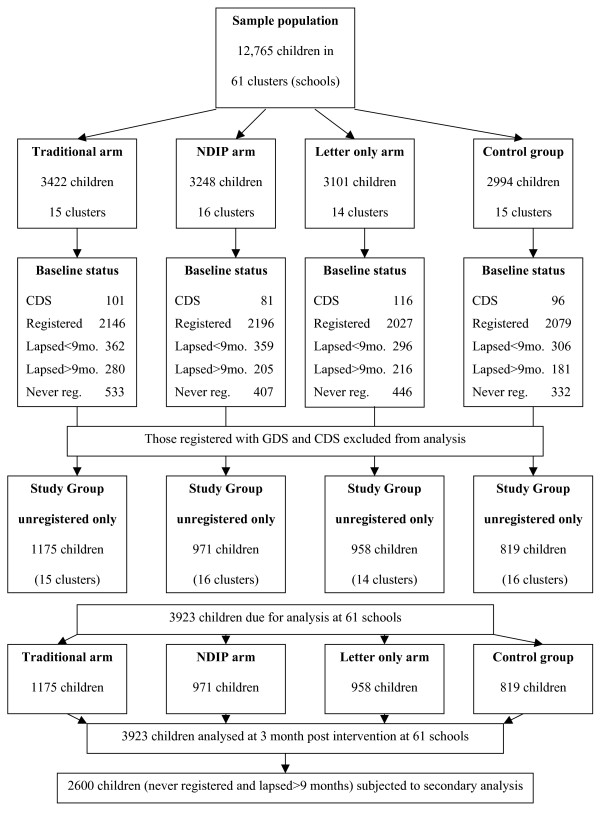
**Illustration of the composition within each arm of the study**.

To evaluate the relative effect of this pre-existing Lothian and Fife system, which is referred to as 'Traditional' throughout the paper, in increasing registration rates in General Dental Practice and compare this with the newly introduced NDIP a study was designed to compare changes in registration rates in General Dental Practice in three groups of 12–13-year-old children (Figure 1) who had been identified as being unregistered against a control group in order to test the null hypotheses listed below:

a) There is no difference between the two inspection methods ('Traditional' and NDIP) in terms of changing registration,

b) The 'Traditional' school screening programme (inspection plus letter) confers no additional benefit on increasing dental registration to sending a letter alone,

c) Sending home letters prompting registration to children confirmed as being unregistered, without conducting a dental inspection, has no effect on dental registration levels.

The main outcome was whether or not children who were unregistered at baseline had become registered 3 months later. Unregistered children fall into two categories, those who have never been registered and those whose previous registration has lapsed. As by the age of 12 these two subgroups within the unregistered population may behave differently regarding registration a secondary analysis was conducted splitting the two groups.

## Methods

The study was designed as a cluster randomised, controlled trial in which the unit of randomisation was the school. In such a trial, the power to detect effects of the interventions depends on both the number of schools and the numbers of children per school, and is a complex function of these and the between- and within-school variation in effect sizes. However the relatively large number of children per school, (range 86–275) meant that the power was likely to be dominated by the number of schools and the variation between schools in the magnitude of the intervention effects. A sample size of approximately 40 schools would be expected to have 80% statistical power to detect a mean difference between intervention and control groups of about one standard deviation in between-school variation. Good power to detect a true mean difference of 10% in take-up rates would require a variation of less than 10% between schools in mean take-up rates resulting from school-specific factors not explained by measurable factors such as deprivation.

For the purpose of this study, first year students in all state secondary schools (n = 61) in Lothian and Fife were identified (12,765 12–13-year-old children) in 2004. All the schools in the sample agreed to participate in the study and the study was approved by the local research ethics committee.

After stratifying for size of school and range of social deprivation using the Scottish Index of Multiple Deprivation [6], the schools were randomly allocated using a computer-generated sequence to one of four groups by the study statistician blinded to the interventions that each would receive which was not revealed until completion of analysis. The four groups and descriptions of the interventions that they received are shown in table 1. Figure 1 illustrates the composition within each arm of the study.

**Table 1:** Number of schools in each group

Group	Intervention	Description of Intervention	No. of schools
1	'Traditional'	Inspections conducted against a checklist of treatment need criteria. Personalised letter for every child, tailored to the confirmed registration status of the child prompting registration where necessary was sent home via the child in sealed, personally addressed envelopes with a list of local GDPs accepting NHS child patients.	15
2	Letter only	As above, but no dental inspection conducted.	14
3	NDIP	Inspections conducted against a checklist of treatment need criteria. Personalised letter for every child – not tailored to the confirmed registration status of the child – was sent home via the child in sealed, personally addressed envelopes with a list of local GDPs accepting NHS child patients.	16
4	Control	Children in the schools allocated to this group received neither a dental inspection nor a letter home until after the end of the study.	16

Following accepted standards in Scotland, in both groups where the children were dentally inspected (1&3) pre-inspection letters were sent home to the parents explaining that their child was to be dentally inspected at school and offering the chance for them to request that their child be exempted from inspection. Children were also at liberty to refuse a dental inspection on the day. A team of 4 community dental officers conducted all inspections starting in February 2004 following protocols according to the allocated group for each school ('Traditional' or NDIP).

Immediately prior to the interventions, the registration status of each child was identified by electronically matching the school lists obtained from the Lothian and Fife Education departments against the dental registration database (Management and Dental Accounting System – MiDAS) held by Dental Practitioner Services within the Common Services Agency of the NHS in Scotland.

Each child was categorised as registered, lapsed or never registered. The time since registration had lapsed was also recorded. The relevant CDS treatment databases were searched and any children in the "unregistered group" found to be under treatment with the CDS in Lothian or Fife were excluded from further analysis.

Only those originally identified as being unregistered (i.e. lapsed or never registered) were analysed. Three months following the interventions changes in registration status were investigated. A further analysis was included to investigate for differences in children who had never been listed as registered with an NHS GDP and those whose had been at one time registered (lapsed more than 9 months). This period was chosen to allow for those individuals whose lapse in registration was only "temporary" and who intended to maintain their registration with the GDS. Also those who had been lapsed for more than 9 months as 2 years would have passed since their last dental inspection which is the maximum recommended period between routine dental check-ups.

Significance tests and confidence intervals for effect sizes were calculated by multilevel modelling using MlwinN software, which allowed the inclusion of predictors of registration rates at both the individual subject level and the school level, and also took appropriate account of the different numbers of children in each school.

## Results

At baseline, of the total S1 population in Lothian and Fife (n = 12,765) two thirds were registered (n = 8448) and, of the remaining third, 394 were receiving treatment from the CDS. Excluding these children left 3923 in the primary analysis.

Table 2 summarises the findings as to whether or not children who were unregistered at baseline had become registered 3 months later.

**Table 2:** Proportion in each study group who had registered after 3 months

Group		No. in group	% registered
1	'Traditional'	1175	14.0
2	Letter only	971	18.1
3	NDIP	958	15.8
4	Control	819	15.8

The registration levels in both the 'Traditional' screening and the NDIP inspection groups rose 3 months post inspection (14% and 15.8% respectively) but were not significantly different from one another or the control group which rose by 15.8% (p > 0.05). The group who were sent a letter home but were not inspected also has a rise in registration levels of 18.1% which was not significantly different from either of the groups who were inspected or the control group (p > 0.05).

**Figure 2:**
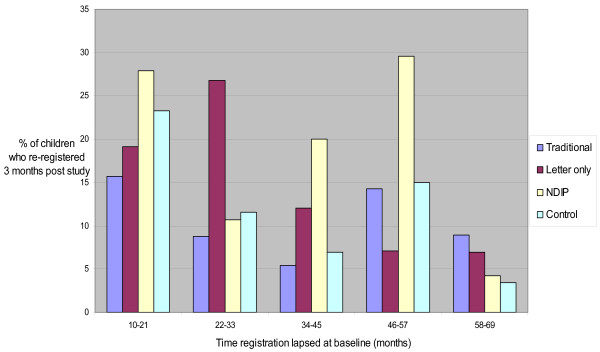
**Re-registration rates of those "lapsed more than 9 months" by length of time lapsed at baseline**.

In the multi-level modelling, among the children who were unregistered at baseline, but who had previously been registered, the most significant predictor of registration at 3 months was the length of time they had been lapsed (P < 0.001), while other predictors tested at pupil level (deprivation score) or school level (region, mean deprivation score, number of pupils in year) were not significant. Figure 2 illustrates the findings for those children who had previously been registered but had lapsed. It shows the downward trend in registration rates with length of time lapsed.

In the 3,923 children who were not registered at the start of the study, 1,323 had previously registered and had lapsed for less than 9 months; these were treated as registered as previously described. Therefore, the analysis in this study was completed on a total of 2,600 children within 61 clusters (schools). Of these, 882 had been lapsed for more than 9 months and 1,718 had never been registered.

The final multilevel model included length of time lapsed as a pupil-level predictor together with study group as the school-level predictor, and the following null hypotheses were tested by comparing study groups:

1. There is no difference between the two inspection methods ('Traditional' and NDIP) in terms of changing registration (Group 1 compared with Group 3),

2. The 'Traditional' school screening programme (inspection plus letter) results in no more dental registration than sending a letter alone (Group 1 compared with Group 2),

3. Simply sending home personalised letters prompting registration (without conducting a dental inspection) has no effect on dental registration levels (Group 2 compared with Group 4).

No statistically significant differences were found to reject the null hypotheses.

**Figure 3:**
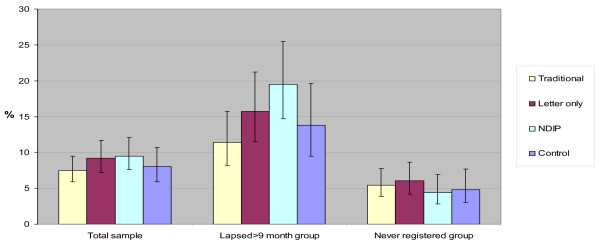
**Increase in registration rate by intervention and previous registration status**.

The secondary analysis looked at differences in the responses to the interventions between those who were once registered (lapsed) and those who were never registered (Table 3). Among those who had never registered there was no significant difference in registration rates in any of the 4 study groups which ranged between 4.4 and 6.1%. However, in those who had previously been registered there were statistically significant differences in the registration rates between those who had a 'traditional' screening (11.4%) and those who were inspected under the NDIP method (19.5%) (p < 0.05). This is illustrated in Figure 3.

**Table 3:** Proportion who had registered after 3 months who at baseline were lapsed or never registered

	Lapsed more than 9 months			Never Registered			Sig. Between Lapsed and Never Registered
Group	No. who registered	No. in group	% registered (CI)	No. who registered	No. in group	% registered (CI)	

1	32	280	11.4* (8.2–15.7)	29	533	5.2 (3.8–7.7)	p < 0.005
2	34	216	15.7 (11.5–21.2)	27	446	6.1 (4.2–8.7)	p < 0.001
3	40	205	19.5* (14.7–25.5)	18	407	4.4 (2.8–6.9)	p < 0.001
4	25	181	13.8 (9.5–19.6)	16	332	4.8 (3.0–7.7)	p < 0.001

* grp 1 vs grp 2 p < 0.05

## Discussion

This study found that there was no significant increase in registration among children who were not registered with a GDP at baseline in any of the four groups. Neither of the two inspection methods ('Traditional' and NDIP) nor a letter sent home to unregistered children prompted statistically significant increases in registration. The findings of our study are in line with the recent large scale study in England who also found that none of their similar interventions were successful in increasing registration rates [4]. The previous studies which showed an increased registration rate following school inspections both involved detailed clinical reports to the parents/guardians and intensive follow up of non-responders [1,7].

Neither of these studies discussed any difference between "lapsed" and "never registered" in the unregistered group. When the two groups were compared in this study there was a significant difference in these two groups of children, with a higher level of re-registration in the group who had lapsed in all 4 groups. In these lapsed children, the time since they lapsed was the strongest predictor of re-registration (Figure 2) however there was no demonstrable difference in re-registrations between the control group and any of the interventions.

One of the unique features of this study is that the use of the MiDAS database allowed follow-up of children's registration status not only within Lothian and Fife but across the whole of Scotland. Since migration out of Scotland is very low in the 0–15 age group [8] it is unlikely that loss to follow-up in this study is of any significance. It is possible that the never registered group had a higher proportion of children who had never had any dental problems and whose parents were therefore not persuaded of the need to register. Additionally, it is not known whether any of the "unregistered" children were under private dental care, though this is unlikely in Scotland as NHS treatment for children is free and at the time of this study the majority of dental practices were accepting child patients under the NHS. More research would be needed in order to confirm the proportion of children under private dental care.

It should be noted that this study was conducted using a cohort of 12–13-year-old children and the findings may not be found within other age groups. This age group however has been identified as exhibiting an increase in the proportion of untreated dental decay [9] and it has been suggested that one possible cause of this worrying decline in dental health is some children failing to access dental services once they leave primary school [10]. First year children in secondary school are no longer routinely inspected in Scotland, and it was deemed important therefore to explore possible options to improve registration rates in this group.

Accuracy in determining registration status has been reported to be problematic. Early studies in this area relied either on parental feedback via a questionnaire to determine registration status [1,3], involvement of the GDP in sending lists to the CDS [7] or on a hand search of dental practice records [11]. Tickle et al [12], used an automated system but reported that making the data sets compatible was found to be time consuming and inappropriate for routine use. The current study used a computer programme devised by the Practitioner Services Department of the Scottish Common Services Agency which used the concept of "probability" matching. This compared various fields in the two matched databases (surname, forename and date of birth) while allowing for possible variations in spelling (e.g. McDonald/MacDonald) or chosen forename e.g. James/Jamie. This system was validated by an internal audit within Fife CDS involving a hand search of GDP records and was shown to be approximately 95% accurate in identifying individual children (Logan, 2001 personal communication). Approval was obtained from the relevant data protection officers when this protocol was introduced in Lothian CDS in 1995. It is uncertain however that such approval would now be obtained given the stricter application of data protection guidelines in recent years. This unique system allowed secondary analysis based upon the time since last registration, giving the opportunity to look at differences in children had never been registered and those who had lapsed from previous registration. There was a significant difference in registration levels in these two subgroups of the unregistered population which suggests that any future studies attempting to influence registration rates should take this potential variable into account.

It is not only difficult to increase dental registration in this age group, but it has also been suggested that registration with a GDP does not equate with a healthy attendance pattern [13]. More importantly perhaps in a study of 8–9-year-olds no difference was found in the dental treatment needs of those who were registered or unregistered [14]. It is possible however, that these findings may be less relevant given the changes that are taking place nationally to remove financial barriers to the provision of preventive care in general dental practice. For example, within Scotland, since the launch of the Action Plan for Dental Services in Scotland [15] many developments have been put in place to promote oral health both at a community and practice levels such as the CHILDSMILE programmes [16].

Recent guidance from the National Screening Committee (NSC) states that three questions need to be answered in relation to school-based dental screening programmes [17]. First, can attendance resulting from screening be improved? This study adds to the evidence currently available to suggest that this is unlikely without committing a large investment in resources to following up individuals post screening. Secondly could treatment rates following referral be improved? This is likely to be best addressed at a national policy level relating to the funding and delivery of NHS GDS services. In Scotland the CHILDSMILE project aims to address both of these points by using the NDIP surveillance system to identify children at most need of care and facilitate and support their continuing care with a local dental practice who have signed up to the 'CHILDSMILE practice' scheme. In addition NDIP data allows health promotion and treatment services to be focused upon those schools identified as having the greatest need. In doing this, Scotland has chosen a different route from England and Wales and it remains to be seen how this will affect the preventive care and treatment provided to Scottish school children. Thirdly, the NSC ask what means might be used to maintain surveillance of dental health of children if the programme were to be abandoned? This is a critical question. Scotland has a rich, historical database gathered from 1987 gathered by the Scottish Heath Boards Dental Epidemiological Programme onto which the NDIP data fits seamlessly. Richards suggested in a recent editorial [18] that greater clarity is required on how to improve the outcomes from the school inspection programme before a decision is made to abandon them. Evaluation of the NDIP interaction with CHILDSMILE will offer valuable information about any potential to improve health gain from school dental inspection programmes.

## Conclusion

In conclusion, neither of the two dental inspection methods nor a letter home to unregistered children resulted in a significant rise in registration rates in 12–13-year-olds compared to a control group of children who received no intervention. Registration with the GDS was more likely in the sub-group of children who had previously registered with a dentist and subsequently lapsed than in those who had never registered. Further study is needed to monitor the effectiveness of other methods of improving treatment rates following referral such as the Scottish CHILDSMILE programme.

## Competing interests

The authors declare that they have no competing interests.

## Authors' contributions

CJC conceived of the study, participated in its design and coordination and drafted the manuscript with GVAT. RE participated in the design of the study and carried out the statistical analysis and commented on the draft manuscript. GVAT participated in the design of the study and drafted the manuscript with CJC. All authors read and approved the final manuscript.

## Pre-publication history

The pre-publication history for this paper can be accessed here:


